# Cement in the heart: a case report of an unusual embolization

**DOI:** 10.1093/ehjcr/ytag564

**Published:** 2026-08-13

**Authors:** Lisa Serafini, Marco Loffi, Dario Brenna, Enrico Passamonti

**Affiliations:** Department of Cardiology, Ospedale di Cremona, ASST Cremona, Cremona 26100, Italy; Department of Cardiology, Ospedale di Cremona, ASST Cremona, Cremona 26100, Italy; Department of Cardiac Surgery, Spedali Civili di Brescia, ASST Spedali Civili di Brescia, Brescia 25123, Italy; Department of Cardiology, Ospedale di Cremona, ASST Cremona, Cremona 26100, Italy

**Keywords:** Atrial mass, Bone cement embolism, Cardiac imaging, Cardiac surgery, Case report

## Summary

A 54-year-old woman with a history of D10 vertebroplasty presented with chest pain, dyspnoea, and fatigue. Chest computed tomography identified a high-density mass in the right atrium (41 × 20 mm). Echocardiography demonstrated interference between the mass and the tricuspid valve, resulting in severe regurgitation and mild right heart dilation.

The patient underwent surgical removal of the mass, which was consistent with embolized bone cement from the prior vertebroplasty, along with tricuspid annuloplasty. The postoperative course was uneventful, with symptomatic improvement and reduction of tricuspid regurgitation to mild.

This case highlights a rare late complication of vertebroplasty due to intracardiac migration of bone cement via the venous system.

A 54-year-old female had undergone percutaneous vertebroplasty procedures for the D-10 vertebra 18 years ago.

She was admitted to our institution for chest pain, accompanied by complaints of cardiac fatigue and dyspnoea over 2 years.

**Figure 1:**
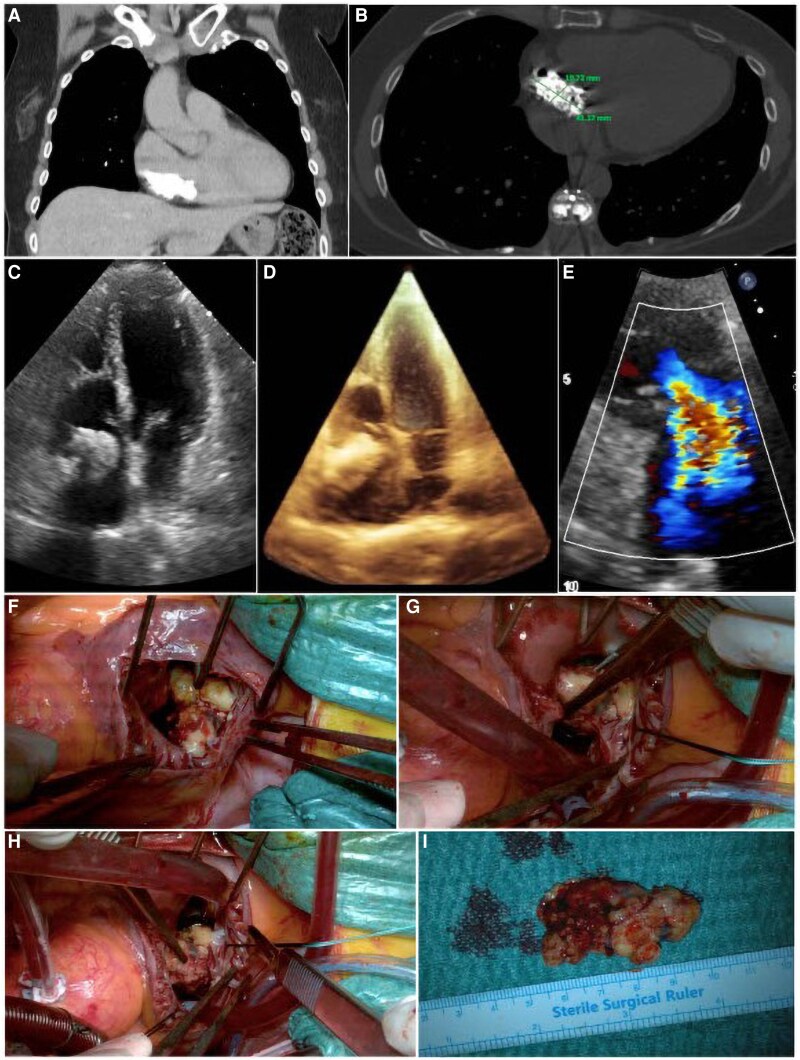
(*A*) Coronal computed tomography section showing high-density mass in the right atrium. (*B*) Transversal computed tomography section showing high density mass in the right atrium, 41 × 20 mm. (*C*, *D*) Echocardiography images that show right atrium mass. (*E*) Echocardiography image showing severe tricuspid regurgitation due to interaction between the mass and the tricuspid valve. (*F–H*) Intra-operative images of mass removal. (*I*) Histological specimen of the excised interatrial mass.

Computed tomography examination of the chest revealed a high-density mass (more than the bone) of 41 × 20 mm (*Figure 1A and B*) occupying the right atrium.

Echocardiography revealed interaction between the mass and the tricuspid valve conditioning leaflets coaptation resulting in severe tricuspid regurgitation (*Figure 1C–E*); mild dilatation of the right ventricle and the right atrium.

The patient was referred to cardiac surgery to remove the mass. Intraoperatively, a though, pale-coloured mass adherent to the lateral wall of the right atrium (between the coronary sinus and the septal tricuspid leaflet) was identified, consistent with orthopaedic material, likely due to embolization of bone cement during prior vertebral surgery (*Figure 1F–I*). The mass was removed and tricuspid valve repair was performed by semirigid-ring annuloplasty. Histological analysis confirmed the presence of amorphous calcific, acellular material.

The patient experienced no procedure-related complications and was discharged on 7th post-operative day, quickly reporting subjective improvement.

Postoperative echocardiography showed an improvement in tricuspid regurgitation, which was graded as mild.

As reported in rare case reports, inadvertent injury to an adjacent vertebral vein during vertebroplasty facilitated retrograde flow of bone cement into the heart via the inferior vena cava and the consequent deposition of the cement in the right atrium.^1^

The data underlying this article are available in the article.

The patient’s data were removed or anonymized. In any case, the patient was informed about the drafting and publication of the article for research purposes and provided informed consent.
